# The Impact of Bodily States on Divergent Thinking: Evidence for a Control-Depletion Account

**DOI:** 10.3389/fpsyg.2017.01546

**Published:** 2017-09-29

**Authors:** Yanyun Zhou, Yifei Zhang, Bernhard Hommel, Hao Zhang

**Affiliations:** ^1^Key Laboratory of Cognition and Personality, Ministry of Education, School of Psychology, Southwest University, Chongqing, China; ^2^Emily Carr University of Art and Design, Vancouver, BC, Canada; ^3^Leiden Institute for Brain and Cognition, Department of Psychology, Leiden University, Leiden, Netherlands

**Keywords:** body and mind, embodied cognition, creativity, divergent thinking, bodily states, roaming

## Abstract

Given previous evidence that bodily states can impact basic cognitive processes, we asked whether such impact can also be demonstrated for creative cognition. In particular, we had participants perform a design improvement task and a consequences imagination task while standing up, walking in a predetermined pattern, or walking freely. Results show better divergent-thinking performance with unconstrained than with constrained walking, and better performance for walking than for standing. A second experiment assessed performance in an alternative uses task and a figural combination task while participants were lying, sitting, or standing. Results showed better performance when standing up than when lying or sitting. Taken altogether, these observations provide evidence for an approach in terms of cognitive-control depletion: the more a bodily activity exhausts control resources, the better divergent thinking can unfold, presumably because reduced top-down control brings more ideas into play.

## Introduction

Most people like to be sitting to think about and solve complex or challenging problems. However, some eminent people are notorious for preferring other bodily postures when seeking original thoughts or solutions. For example, Friedrich Nietzsche had his key insight into the will while he was walking (Nietzsche, 1897; Platt, 1976), Ernest Hemingway was standing up while writing about some soul-stirring characters for his novels (Hotchner, 2005), and Mark Twain was lying in bed to produce his opus magnum (Twain, 2002). Can bodily postures or activities of an individual really affect the generation of creative ideas? If so, which bodily state is the best?

The interest in the interaction between body and mind dates back at least to ancient Plato, who claimed that mind could not be explained by body (Robinson, 1983). More recently, Descartes believed that mind exists independently of the body (Descartes, 2008). And yet, very recent studies provide converging evidence that bodily activities can impact on various cognitive processes including perception (Balcetis and Dunning, 2007), memory (Zajonc et al., 1982; Scott et al., 2001), language comprehension (Olmstead et al., 2009), judgment (Cacioppo et al., 1993; Beilock, 2009), emotion (Strack et al., 1988; Glenberg et al., 2005), and decision making (Borghi and Cimatti, 2010). These findings suggest that at least some cognitive processes are embodied (body-dependent), in the sense the mental/brain processes rely on, or are affected by the physical body (Wilson and Foglia, 2011).

Earlier studies have examined the influence of bodily position (lying, sitting, and standing) on basic cognition (Woods, 1981; Vercruyssen and Simonton, 1988; Vercruyssen et al., 1989). For example, Woods (1981) found that older participants’ visual choice reaction time was significantly shorter in standing than in lying or sitting conditions. However, in a more complex anagram task, Lipnicki and Byrne (2005) found participants performing better when lying down than when standing. Even more important for our purposes, Oppezzo and Schwartz (2014) investigated the effect of sitting and walking on the generation of novel responses in various creativity tasks. Participants performed better in the alternative uses task (AUT) when walking on a treadmill than when sitting down and, in another experiment, better when walking along a predetermined pathway at the university campus than when sitting. Yet another experiment extended these observations to Barron’s symbolic equivalence task, which requires the generation of analogies: performance was better when walking along the predetermined pathway at the campus than when walking on a treadmill or sitting on a chair in a room or in a wheelchair moved across the campus by a confederate.

Oppezzo and Schwartz’s (2014) study provides rather strong evidence for an important role of bodily states and activities in creative divergent thinking, but the authors do not provide a mechanistic explanation for how the body may impact the mind. In particular, one may think of two possible, not necessarily mutually exclusive accounts to conceptualize the obtained findings. For one, it has been suggested that engaging in one activity may provide metaphors that make concepts and knowledge from a superficially dissimilar domain mentally available (Landau et al., 2010; IJzerman and Koole, 2011). Along these lines, it has been shown that performance in divergent thinking tasks benefits from the enactment of presumably creativity-related metaphors, like postures involving both hands (thought to facilitate the thinking in terms of “on the one hand, on the other”), taking place within or outside a box, or walking freely or along a fixed rectangular path (Leung et al., 2012). From this conceptual-metaphor approach, the findings of Oppezzo and Schwartz (2014) could be taken to reflect the metaphoric relationship between moving around physically and moving around mentally, as needed in divergent thinking, which would lead to the facilitation of the latter by engaging in the former.

For another, however, there is increasing evidence that divergent thinking benefits from the depletion of cognitive-control resources. Increasing evidence suggests that people can engage in different control styles, ranging from extreme persistence (reflecting strong competition of alternative representations guided by strong top-down guidance from the current goal) to extreme flexibility (reflecting weak competition of alternative representations with little top-down impact; for an overview, see Hommel, 2015). This implies that impairing top-down control should drive the control style toward flexibility, which in turn should benefit performance in divergent thinking tasks. While extreme forms of physical exhaustion tend to impair various forms of creative thinking in non-athletes (Colzato et al., 2013), there is indeed increasing evidence that milder forms of overloading top-down control can indeed facilitate divergent thinking. For instance, engaging in a control-hungry cognitive-conflict tasks has been found to improve performance in an AUT (Radel et al., 2015) and aging, a condition that is known to weaken top-down control, is associated with improvements in a number of tasks that require novel responses (for a summary, see Amer et al., 2016). Along the same lines, bilingualism—which has been shown to increase top-down control (Bialystok and Craik, 2010)—is associated with improved convergent thinking but impaired divergent thinking (Hommel et al., 2011).

To get more insight into the mechanisms underlying the impact of bodily postures and activities on divergent thinking, we conducted two pairs of experiments with altogether four different divergent-thinking tasks. In Experiment 1, we sought to conceptually replicate and extend the findings of Oppezzo and Schwartz (2014) by using a design improvement task (DIT) in Experiment 1A and a consequences imagination task (CIT) in Experiment 1B. Similar to Oppezzo and Schwartz’s (2014) conditions of sitting and constrained walking, we employed standing, constrained walking, and unconstrained walking (roaming) conditions (Leung et al., 2012), which allowed us to directly compare performance under all three conditions. We expected to replicate and extend Oppezzo and Schwartz’s (2014) observation of better divergent thinking in the constrained-walking (non-roaming) condition than in the non-walking condition, as well as Leung et al.’s (2012) finding of better divergent thinking in the unconstrained than in the constrained walking condition.

Experiment 2 was designed to get one step further by testing predictions from the conceptual-metaphor approach against the control-depletion approach. Given the both approaches could accommodate the findings from Experiment 1, we were interested to compare conditions that would allow to disentangle the two. To do so we had participants sitting down, lying down, or standing up—conditions that should be comparable from a conceptual-metaphor point of view (as none of the conditions would include moving) but that put different demands on cognitive control (with standing up being the most exhaustive condition). Accordingly, we were interested to see whether divergent thinking would be comparable across these three conditions or whether standing up would be particularly beneficial. We tested these three conditions by using two different tasks, the AUT (Experiment 2A) and the figural combination task (FCT; Experiment 2B).

## Experiment 1A

### Methods

#### Participants

Sixty-three college students (21 males and 42 females, mean age 21.25 years, range 18–25 years, mean height 1.63 m, range 1.50–1.86 m; mean weight 52.4 kg, range 42–61 kg) were paid for participation. All participants were right-handed, with normal or corrected-to-normal vision, and all wore comfortable flat shoes. None of them had a history of neurological or psychiatric mental problems or a physical disability. The study was approved by the University Human Experiment Ethical Committee and informed written consent was obtained from all participants.

#### Materials

A DIT was employed to assess divergent thinking performance. This task involves real-life problem solving for which novel ideas are required to improve the design of existent objects/devices (e.g., how to design an outdoor chair that can be comfortably sat upon even when wet). Each problem was presented auditorily through a wireless headset, which also served to pick up the vocal responses. The length of each problem was about 13 or 14 Chinese characters. A wireless mouse was held in the right hand to switch to the next problem.

The experiment was conducted in an empty room (7.4 m × 3.5 m). In the standing condition, participants were requested to stand naturally in the center of the room to complete the task. In the roaming condition, participants completed the task while walking freely in the room without constraints in direction or speed. In the non-roaming condition, participants completed the task while walking along an 8-shaped path of 16.8 m length without speed constraints. The Figure-of-8 Walk Test (F8W) has been widely used to investigate walking (Hess et al., 2010; Lowry et al., 2012). During the present experiment, half of the participants were required to continually walk the path in a clockwise direction while the other half were required to walk in a counterclockwise direction.

#### Design

A within-subject research design was used to examine the effect of the three bodily states (standing, constrained walking/non-roaming, unconstrained walking/roaming) on the DIT. To avoid confounds through fatigue and practice, the order of the three conditions was counterbalanced across participants. After each condition, there was a short break of 2 min.

#### Procedure

During each trial, a starting vocal prompt of 500 ms was followed by a problem presented for 3700∼3800 ms. Participants were requested to consider a novel solution to the present problem for 15 s. When the vocal prompt “Please give your answer” was presented at the end of this time period, participants had to report orally one solution which they thought to be the most novel. Then they would start the next trial by pressing the mouse button. To assess whether the walking speed influences the creative process, the speed (the number of steps a participant walked per minute) in the two walking conditions was recorded by the experimenter. Considering that preference of participants’ bodily states may have an impact, participants were asked to rate their preference for each bodily state after the experiment on a 7-point scale ranging from 1 (no preferable) to 7 (very preferable).

### Results

**FIGURE 1:**
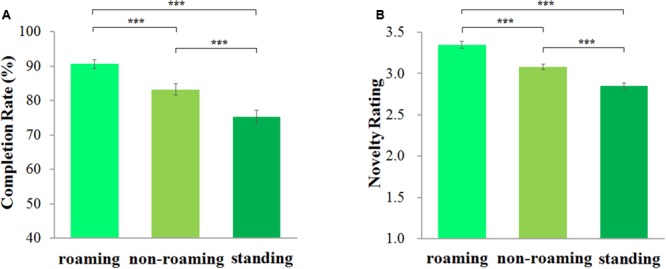
Effect of bodily states on the design improvement task. Completion rate is the mean percentage of solutions participants found in bodily state conditions **(A)**; novelty rating score is the mean of originality scores rated by six raters on a 5-point scale ranging from 1 “not original” to 5 “very original” **(B)**. Error bars are standard errors of the mean. ^∗∗∗^*p* < 0.001.

Completion rates were counted and recorded by the experimenter and the novelty of ideas was rated by six experts on a 5-point scale ranging from 1 (not original) to 5 (very original). All experts that performed the rating of the answers were unaware of the experimental conditions participants were assigned to—which holds for all experiments reported in this article. The inter-rater reliability among the raters reached a Cronbach’s alpha of 0.79. Completion rates and novelty rating scores per condition were analyzed by repeated-measures ANOVAs (SPSS 13.0 for Windows). The results showed a main effect on completion rates, *F*(2,124) = 38.60, *p* < 0.001, η^2^ = 0.384. Further analysis (Bonferroni corrected, as in all following comparisons) showed that the completion rate was significantly higher in the roaming condition (*M* = 90.63%, *SE* = 0.01) than in both the non-roaming condition (*M* = 83.17%, *SE* = 0.02), *p* < 0.001, and the standing condition (*M* = 75.24%, *SE* = 0.02). Also, the completion rate was significantly higher in the non-roaming than that in the standing condition, *p* < 0.001 (**Figure 1A**).

In addition, there was a significant main effect for the novelty rating score, *F*(2,124) = 61.04, *p* < 0.001, η^2^ = 0.496. Further comparisons showed that novelty ratings were higher in the roaming condition (*M* = 3.35, *SE* = 0.04) than in both the non-roaming condition (*M* = 3.08, *SE* = 0.03), *p* < 0.001, and the standing condition (*M* = 2.85, *SE* = 0.04), *p* < 0.001. Also, novelty ratings were higher the non-roaming condition than in the standing condition, *p* < 0.001 (**Figure 1B**).

Walking speed was analyzed with paired *t*-tests. Results showed that there was no significant difference in walking speeds between the roaming (65.04 steps/min) and the non-roaming condition (66.38 steps/min), *t*_(62)_ = 1.396, *p* > 0.1. We also analyzed the impact of participants’ preference of bodily states by computing Pearson correlations between bodily state preference and completion rates and novelty rating scores. Results showed there was no correlation (all two-tailed) between completion rate and bodily state preference (standing, *r* = -0.071, *p* > 0.1; roaming, *r* = -0.062, *p* > 0.1; non-roaming, *r* = 0.127, *p* > 0.1). There was also no correlation between novelty rating score and bodily state preference (standing, *r* = -0.108, *p* > 0.1; roaming, *r* = 0.178, *p* > 0.1; non-roaming, *r* = 0.179, *p* > 0.1).

## Experiment 1B

### Methods

#### Participants

The same 63 college students (21 males and 42 females, mean age 21.25 years, range 18–25 years; mean height 1.63 m, range 1.50–1.86 m; mean weight 52.4 kg, range 42–61 kg) tested in Experiment 1A participated in this experiment for pay, they fulfilled the exact same criteria as in Experiment 1A. The study was approved by the University Human Experiment Ethical Committee and informed written consent was obtained from all participants.

#### Materials

These were as in Experiment 1A, except that CIT was employed to assess divergent thinking. It has been considered a classic measure of the creative process and involves both divergent thinking ability and the capacity of imagination (Torrance, 1987; Kim et al., 2006; de Souza et al., 2010; Wei et al., 2014). Participants were asked to imagine the possible consequences of an assumed scenario (e.g., what will happen if people need no sleep?) and were requested to consider possible outcomes of the assumed case and give as many novel ideas as possible. Each problem consisted of nine Chinese characters, which were presented auditorily. In total, there were 10 stimulus trials per condition.

#### Design

The design was similar to Experiment 1A, except for the dependent measure.

#### Procedure

In each trial, a starting vocal prompt of 500 ms, and then a vocal stimulus was presented for 3900∼4000 ms, participants were requested to consider and speak out novel outcomes to an assumed case within 1 min immediately after the present of the stimulus. In this time period, participants had to vocally report as many novel outcomes of the imaginary scenario as possible. After that, next stimulus trial was presented automatically.

### Results

Fluency and flexibility of responses were counted and recorded by the experimenter and novelty was rated by six experts, like in Experiment 1A. Cronbach’s alpha = 0.70. Fluency, flexibility, and novelty rating scores were analyzed by means of repeated-measures ANOVAs. The main effect was significant for fluency, *F*(2,124) = 90.69, *p* < 0.001, η^2^ = 0.594, flexibility, *F*(2,124) = 60.06, *p* < 0.001, η^2^ = 0.492, and novelty, *F*(2,124) = 165.92, *p* < 0.001, η^2^ = 0.728.

**FIGURE 2:**
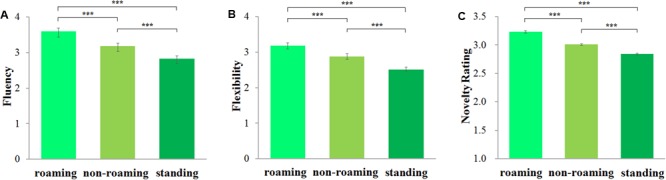
Effect of bodily states on the consequences imagination task. Fluency score is the mean number of responses participants generated in bodily state conditions **(A)**; flexibility score is the mean number of different categories of responses in bodily state conditions **(B)**; novelty rating score is the mean of originality scores by six raters on a 5-point scale ranging from 1 “not original” to 5 “very original” **(C)**. Error bars are standard errors of the mean. ^∗∗∗^*p* < 0.001.

Further comparisons showed that fluency was higher in the roaming condition (*M* = 3.60, *SE* = 0.09) than in both the non-roaming condition (*M* = 3.18, *SE* = 0.08), *p* < 0.001, and the standing condition (*M* = 2.83, *SE* = 0.08), *p* < 0.001. The difference between the non-roaming condition and the standing condition was also significant, *p* < 0.001 (**Figure 2A**). Likewise, flexibility was higher in the roaming condition (*M* = 3.18, *SE* = 0.09) than in both the non-roaming condition (*M* = 2.88, *SE* = 0.08), *p* < 0.001, and the standing condition (*M* = 2.51, *SE* = 0.07), *p* < 0.001. The difference between non-roaming and standing was also significant, *p* < 0.001 (**Figure 2B**). Finally, novelty was higher in the roaming condition (*M* = 3.22, *SE* = 0.02) than in both the non-roaming condition (*M* = 3.01, *SE* = 0.02), *p* < 0.001, and the standing condition (*M* = 2.84, *SE* = 0.01), *p* < 0.001. The difference between non-roaming condition and standing was also significant, *p* < 0.001 (**Figure 2C**).

Further analyses showed that there was no significant difference between the walking speeds in the roaming condition (64.78 step/min) and the non-roaming condition (65.83 step/min), *t*_(62)_ = 1.032, *p* > 0.1, suggesting that walking speed did not moderate the effects. Likewise, bodily state preference did not correlate with fluency, flexibility, or novelty ratings: fluency (standing, *r* = 0.100, *p* > 0.1; roaming, *r* = 0.018, *p* > 0.1; non-roaming, *r* = -0.022, *p* > 0.1); flexibility (standing, *r* = 0.101, *p* > 0.1; roaming, *r* = 0.044, *p* > 0.1; non-roaming, *r* = 0.09, *p* > 0.1); novelty (standing, *r* = -0.211, *p* > 0.1; roaming, *r* = -0.1, *p* > 0.1; non-roaming, *r* = 0.082, *p* > 0.1).

### Summary

Experiments 1A and 1B served to conceptually replicate and extend the findings of Oppezzo and Schwartz (2014) by using a CIT and a DIT, and by adding an unconstrained walking condition as used by Leung et al. (2012). Even for these new tasks, the results successfully replicated the Oppezzo and Schwartz’s (2014) observation of better divergent-thinking performance with the constrained walking than with non-walking, suggesting that the impact of walking widely generalizes. The results also provide a conceptual replication of Leung et al.’s (2012) finding that unconstrained walking facilitates divergent thinking more than constrained walking does.

## Experiment 2A

### Method

#### Participants

Sixty-one new college students participated in this experiment. One was excluded due to the mean of her creative idea scores deviating more than 3 standard deviations from the participants’ mean. Thus, the final sample comprised 60 subjects (13 males and 47 females, mean age 20.98 years, range 18–24 years). The study was approved by the University Human Experiment Ethical Committee and informed written consent was obtained from all participants.

#### Materials

A classic AUT (Christensen et al., 1960; Guilford et al., 1978) was employed. In this experiment, a stimulus item was name of an object (e.g., pencil). Participants were requested to speak out as many unusual uses of the object as they could. Thus there were 10 stimulus items per condition of the bodily state.

#### Design

A within-subject design was used to examine the effect of three bodily states (lying, sitting, and standing) on AUT. To avoid artifacts from fatigue and practice effects, the order of the three conditions was counterbalanced across participants. In the lying condition, participants were lying on a bed (210 cm × 90 cm × 40 cm) with their hands being put comfortably in parallel with their body. A trestle (150 cm × 30 cm × 104 cm) was placed across the bed, holding a computer screen above the participant’s eyes. In the sitting condition, the participants were seated in a chair (43 cm height) with their feet on the floor and their hands on the lap, facing a computer screen placed on a table (78 cm height) in front of the participants. In the standing condition, participants stood upright quietly with their feet held naturally on the floor and the arms hanging naturally, and a computer screen was placed on a table (150 cm height) in front of the participants. In all conditions, the distance between the participants’ eyes and the computer screen was 60 cm. Participants held a computer mouse in their right hand and pressed a mouse button to indicate their responses. Trials were presented continuously in each condition, but there were short breaks of 2 min between conditions.

#### Procedure

Before the experiment, participants practiced the tasks to fully familiarize themselves with the experimental instruction and procedure. During the experiment, stimulus items were presented on the computer screen one by one. In the beginning of each trial, a fixation mark was presented for 0.5 s, then the name of an object appeared in the center of the screen and lasted for 1 min. Within the period of 1 min participants were requested to speak out as many unusual uses of the object as they could. Then next stimulus trial was presented automatically. Each condition contained 10 trials.

### Results

As usual, the answers in the three conditions were scored according to fluency (total number of uses of each object), flexibility (number of different categories of uses), and novelty by five experts on a 5-point scale ranging from 1 “not original” to 5 “very original.” The inter-rater reliability among the raters reached a Cronbach’s alpha of 0.76.

**FIGURE 3:**
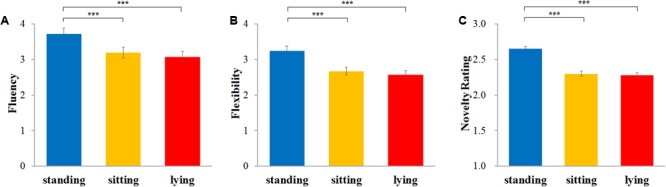
Effect of bodily states on the alternative uses task. Fluency score is the mean number of responses generated for an object/device in bodily state conditions **(A)**; flexibility score is the mean number of different categories of responses in bodily state conditions **(B)**; novelty rating score is the mean of originality scores rated by five raters on a 5-point scales ranging from 1 “not original” to 5 “very original” **(C)**. Error bars are standard errors of the mean. ^∗∗∗^*p* < 0.001.

Fluency, flexibility, and novelty scores were analyzed by means of repeated-measures ANOVA with condition as within-participants variable. Significant main effects were obtained for fluency, *F*(2,118) = 34.37, *p* < 0.001, η^2^ = 0.368, flexibility, *F*(2,118) = 52.14, *p* < 0.001, η^2^ = 0.469, and novelty, *F*(2,118) = 78.06, *p* < 0.001, η^2^ = 0.570. Further comparisons showed that fluency was significantly higher in the standing condition (*M* = 3.71, *SE* = 0.17) than in both the lying condition (*M* = 3.08, *SE* = 0.15), *p* < 0.001, and the sitting condition (*M* = 3.20, *SE* = 0.15), *p* < 0.001, while the difference between lying and sitting was not significant, *p* > 0.1 (**Figure 3A**). Flexibility was significantly higher in the standing condition (*M* = 3.25, *SE* = 0.14) than in both the lying condition (*M* = 2.57, *SE* = 0.11, *p* < 0.001), and the sitting condition (*M* = 2.67, *SE* = 0.11), *p* < 0.001, while the difference between lying and sitting was not significant, *p* > 0.1 (**Figure 3B**). Finally, novelty was significantly higher in the standing condition (*M* = 2.65, *SE* = 0.03) than in both the lying condition (*M* = 2.28, *SE* = 0.04), *p* < 0.001, and the sitting condition (*M* = 2.30, *SE* = 0.04) *p* < 0.001, while the difference between lying and sitting was not significant, *p* > 0.1 (**Figure 3C**).

Further analyses showed no correlation between bodily preference and any of the creativity scores: fluency (lying, *r* = 0.03, *p* > 0.1; sitting, *r* = 0.16, *p* > 0.1; standing, *r* = -0.16, *p* > 0.1); flexibility (lying, *r* = 0.06, *p* > 0.1; sitting, *r* = 0.18, *p* > 0.1; standing, *r* = -0.19, *p* > 0.1); novelty (lying, *r* = 0.14, *p* > 0.1; sitting, *r* = -0.04, *p* > 0.1; standing, *r* = 0.01, *p* > 0.1).

## Experiment 2B

### Method

#### Participants

The same 61 participants tested in Experiment 2A participated in this experiment. Two of them were excluded because they failed to completely follow the instruction to do stimulus task, thus the final sample comprised 59 subjects (13 males and 46 females, mean age 21.00 years, range 18–24 years). All participants were wearing comfortable flat shoes in the experiment. They were paid for their participation. The study was approved by the University Human Experiment Ethical Committee and informed written consent was obtained from all participants.

#### Materials

A FCT was employed. It has been widely used to investigate creative imagination (Finke, 1990; Verstijnen et al., 1998; Abraham et al., 2005, 2007; Chiu, 2012). In this experiment, per stimulus item consisted of three different geometric figures randomly chosen from the 15 figures like Finke’s experiment (Finke, 1990). There were ten stimulus items each experimental condition. The participants were required to combine them to form an object/device in a specified category.

#### Design

The design was as in Experiment 2A, except for the task.

#### Procedure

The procedure was as in Experiment 2A, with a few exceptions. In the beginning of each trial, a 0.5-s fixation mark was followed by a prompt to indicate randomly one of six categories and a triplet of geometric figures. Participants were asked to combine the three geometric components into an object/device belonging to the given category. These geometric components were allowed to vary their size, materials, colors, and orientation, but they were not allowed to change in shape. In addition, all geometric components in a trial had to be used together and could not be used separately for a different objects or devices. Participants were asked to press the button as soon as possible when an idea would come to mind, and then write a brief statement and draw a sketch of the object/device they were thinking of. If participants could not report or describe an object/device, the display would disappear at the end of 2 min, and the next trial would commence.

### Results

**FIGURE 4:**
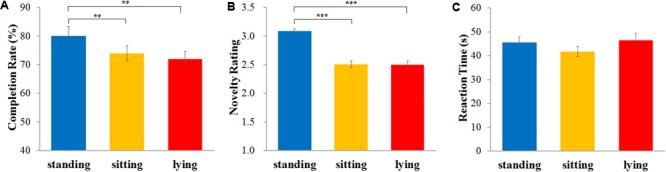
Effect of bodily states on the figural combination task. Completion rate is the mean percentage of products participants generated in bodily state conditions **(A)**; novelty rating score is the mean of originality scores rated by four raters on a 5-point scale ranging from 1 “not original” to 5 “very original” **(B)**. Reaction time is the mean time of a product participants generated in bodily state conditions **(C)**. Error bars are standard errors of the mean. ^∗∗^*p* < 0.01. ^∗∗∗^*p* < 0.001.

Completion rates were counted and recorded by experimenter and reaction times were recorded automatically, and novelty of the objects/devices was rated by four experts on a 5-point scale ranging from 1 (not original) to 5 (very original). Inter-rater reliability reached Cronbach’s alpha = 0.71. A repeated-measures ANOVA for completion rate yielded a significant main effect of condition, *F*(2,116) = 6.59, *p* < 0.005, η^2^ = 0.102. Further comparisons showed that completion rate were significantly higher in the standing condition (*M* = 80.00%, *SE* = 0.03) than in both the lying condition (*M* = 71.86%, *SE* = 0.03), *p* < 0.01, and the sitting condition (*M* = 74.07%, *SE* = 0.03), *p* < 0.01, while there was no significant difference between lying and sitting, *p* > 0.1 (**Figure 4A**).

The ANOVA of the novelty ratings also showed a significant main effect, *F*(2,116) = 39.76, *p* < 0.001, η^2^ = 0.407. Further comparisons confirmed that the novelty ratings were significantly higher in the standing condition (*M* = 3.09, *SE* = 0.04) than in both the lying condition (*M* = 2.50, *SE* = 0.07), *p* < 0.001, and the sitting condition (*M* = 2.51, *SE* = 0.06), *p* < 0.001, while the difference between lying and sitting was not significant, *p* > 0.1 (**Figure 4B**).

An ANOVA of the reaction times showed no significant main effect, *F*(2,116) = 2.05, *p* > 0.1, η^2^ = 0.034 (**Figure 4C**). The bodily state preference did not correlate significantly with the completion rate (lying, *r* = -0.12, *p* > 0.1; sitting, *r* = -0.04, *p* > 0.1; standing, *r* = -0.06, *p* > 0.1) or novelty (lying, *r* = -0.12, *p* > 0.1; sitting, *r* = 0.04, *p* > 0.1; standing, *r* = 0.02, *p* > 0.1).

### Summary

Experiments 2A and 2B provide systematic convergent evidence that participants generate more and newer ideas when standing up than when either lying down or sitting, while the lying or sitting did not make a difference.

## General Discussion

The present study investigated the role of bodily states in creative idea generation. Experiment 1 conceptually replicated and extended previous evidence suggesting that divergent thinking benefits more from constrained walking than from non-walking, and more from unconstrained than from constrained walking. As pointed out in the introduction, this could be due to the possibility that walking provides access to metaphorically related knowledge, which in turn might support mentally moving through one’s memories—as needed for creative thinking. Alternatively, standing, constrained walking, and unconstrained walking can be taken to represent conditions of increasing cognitive difficulty, which in turn would imply increasing depletion of cognitive-control resources. To test these possibilities against each other, we had the participants of Experiment 2 to take on three postures that are likely to differ in their reliance on control resources (with standing up and keeping balance being the most depleting condition) but not in their metaphorical relationship to mental movements. The outcome is clear-cut, given that both tasks being tested and all measures being assessed showed significantly better divergent thinking when standing up than when either sitting or lying. While this observation does not necessarily rule out the conceptual-metaphor approach, it cannot be explained by it either. We thus take the findings from Experiment 2 as support for the control-depletion approach, the more so as this approach also provides a parsimonious account for the findings from Experiment 1.

Indeed, walking (and unconstrained walking in particular) consumes cognitive resource, so that less resource is devoted to other tasks (Lacour et al., 2008). While this would be unlikely to be beneficial for other tasks that rely on a strong degree of focusing themselves, a lack of focusing has been shown to be beneficial for problem solving (Mendelsohn and Griswold, 1966; Ansburg and Hill, 2003; Dorfman et al., 2008). This also fits with observations that patients with attention deficit hyperactivity disorder often exhibit high fluency, flexibility, and originality in the AUT and high creative scores in the creative achievement questionnaire (White and Shah, 2006, 2011). Authors have suggested that this may be because defocusing or reducing top-down control reduces selectivity and brings into play a greater variety of possible responses (Vartanian et al., 2007; Dorfman et al., 2008; Hommel, 2012), which for instance can help to overcome functional fixedness (Finke et al., 1992; Howard-Jones and Murray, 2003) and increase the probability to form more remote associations and more original ideas (Finke et al., 1992; Martindale, 1995; Hutton and Sundar, 2010). Therefore, defocused attention has been claimed to be conducive to the generation of novel ideas (Suler, 1980; Finke et al., 1992; Martindale, 1995, 1999; Smith, 1995; Gruszka and Necka, 2002; Ansburg and Hill, 2003; Howard-Jones and Murray, 2003; Glazer, 2009; Zabelina and Robinson, 2010; Jarosz et al., 2012). According to Hommel (2015), creative thinking requires the identification of representations in memory that fit with the current search template, such as the features of the sought-for object in a creativity task. Each representation receives top-down support to the degree that it meets the criteria of the search template and it inhibits alternative representations. Hommel (2012) suggests that the degree of both top-down support and lateral inhibition relies on cognitive resources, which implies that a depletion of resources reduces top-down support and lateral inhibition. While convergent thinking is likely to suffer from this condition, brainstorming-like divergent thinking is likely to benefit (as long as some degree of top-down regulation is present), as the lack of lateral inhibition facilitates jumping from one representation to another (Hommel, 2012). Our present findings are consistent with, and provide converging evidence for this claim. Cognitive-control demands are likely to increase from sitting to standing up, to constrained walking, and finally to unconstrained walking. While most of these activities are assumed to operate unconsciously, there is nevertheless evidence suggesting that they are sensitive to manipulations of cognitive load. For instance, elderly participants show impairments in walking when being asked to memorize items in working memory at the same time (Li et al., 2001). This suggests that even highly automatized activities rely on cognitive resources and, thus, can suffer from depletion. There is also evidence that free choice requires more cognitive resources than constrained choice (Berlyne, 1957), which accounts for our finding that unconstrained walking showed the largest effects in the present study. Our findings call for a systematic assessment of the resource demands of activities, which would allow for a more objective and systematic prediction of the expected benefits in divergent thinking. For the time being, we thus restrict ourselves to the conclusion that at least some sort of relationship exists between the resource demands of human activities and the performance in brainstorming-like tasks.

## Author Contributions

YaZ and HZ contributed to the study design. YaZ performed the data collection and analyses. YaZ and HZ interpreted the data. YaZ, YiZ, BH, and HZ contributed to the paper writing. All authors approved the final version of the manuscript for submission.

## Conflict of Interest Statement

The authors declare that the research was conducted in the absence of any commercial or financial relationships that could be construed as a potential conflict of interest.
